# Glucocorticoid induced bone disorders in children: Research progress in treatment mechanisms

**DOI:** 10.3389/fendo.2023.1119427

**Published:** 2023-04-04

**Authors:** Junying Hua, Jianping Huang, Gang Li, Sien Lin, Liao Cui

**Affiliations:** ^1^ Guangdong Provincial Key Laboratory of Research and Development of Natural Drugs, School of Pharmacy, Guangdong Medical University, Zhanjiang, China; ^2^ Department of Prosthodontics, Yonsei University College of Dentistry, Seoul, Republic of Korea; ^3^ Musculoskeletal Research Laboratory, Department of Orthopaedics & Traumatology, Faculty of Medicine, Prince of Wales Hospital, The Chinese University of Hong Kong, Hong Kong, Hong Kong SAR, China; ^4^ Stem Cells and Regenerative Medicine Laboratory, Li Ka Shing Institute of Health Sciences, The Chinese University of Hong Kong, Hong Kong, Hong Kong SAR, China; ^5^ Orthopaedic Center, Affiliated Hospital of Guangdong Medical University, Guangdong Medical University, Zhanjiang, China

**Keywords:** growth hormone, growth plate, bone growth suppression, glucocorticoid (GC), chondrocyte 3

## Abstract

Long-term or supra-physiological dose of glucocorticoid (GC) application in clinic can lead to impaired bone growth and osteoporosis. The side effects of GC on the skeletal system are particularly serious in growing children, potentially causing growth retardation or even osteoporotic fractures. Children’s bone growth is dependent on endochondral ossification of growth plate chondrocytes, and excessive GC can hinder the development of growth plate and longitudinal bone growth. Despite the availability of drugs for treating osteoporosis, they have failed to effectively prevent or treat longitudinal bone growth and development disorders caused by GCs. As of now, there is no specific drug to mitigate these severe side effects. Traditional Chinese Medicine shows potential as an alternative to the current treatments by eliminating the side effects of GC. In summary, this article comprehensively reviews the research frontiers concerning growth and development disorders resulting from supra-physiological levels of GC and discusses the future research and treatment directions for optimizing steroid therapy. This article may also provide theoretical and experimental insight into the research and development of novel drugs to prevent GC-related side effects.

## 1 Introduction

**Figure 1:**
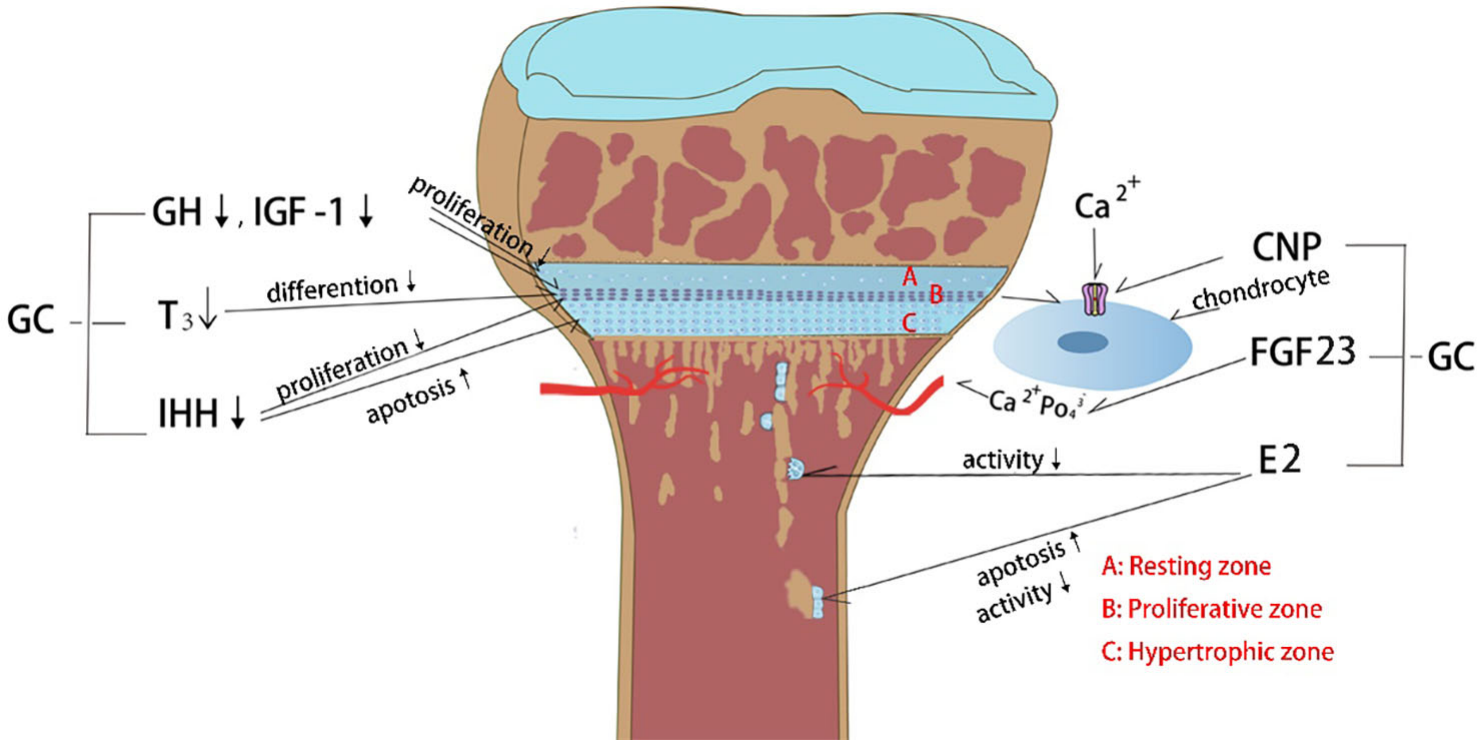
Effects of glucocorticoid (GC) on endocrine hormones and cytokines. (1) GC decreases growth hormone (GH) and insulin growth factor-1 (IGF-1) levels which in turn inhibits the chondrocyte proliferation. (2) GC affects thyroid hormone secretion and further inhibits the chondrocyte hypertrophy. (3) GC decreases Indian hedgehog factor (IHH) secretion and then inhibits chondrocyte proliferation and increases chondrocyte apoptosis. (4) GC constrains C-type natriuretic peptide (CNP) production, which controlling the entry of calcium ion into chondrocytes to stimulate growth. (5) GC causes hypocalcemia through upregulation of fibroblast growth factor 23 (FGF23) expression in bone and plasma, which in turn inhibits chondrocyte proliferation. (6) GC inhibits estrogen (E2) secretion, causing decreased osteoclast activity and increased osteoblast apoptosis.

**Figure 2:**
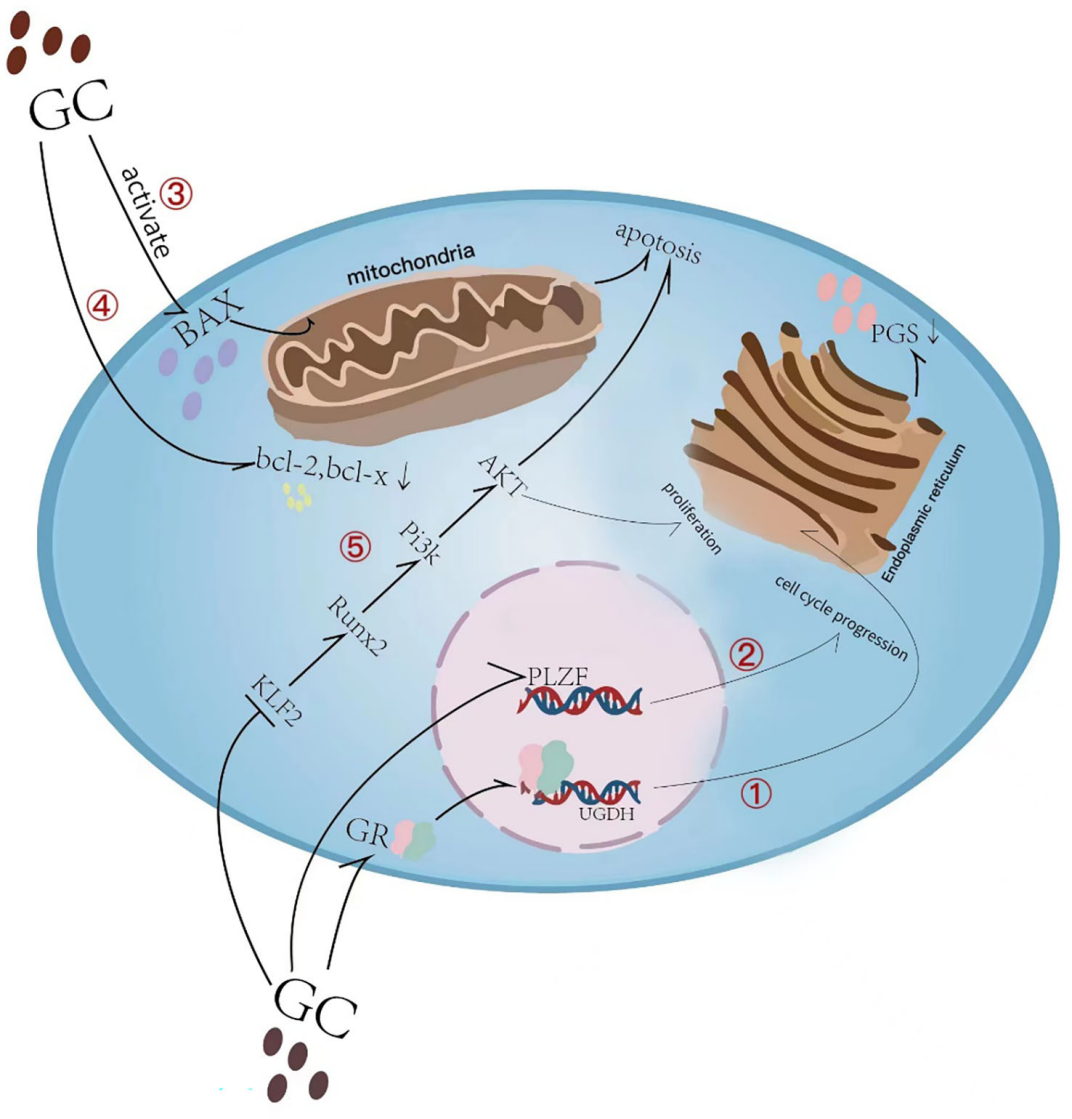
Overview of glucocorticoid-induced chondrocyte apoptosis and matrix synthesis reduction. (1) Glucocorticoid (GC) induces direct binding of glucocorticoid receptor (GR) to the uridine diphosphate glucose dehydrogenase (UGDH) promoter in chondrocytes and suppresses UGDH gene expression, a change that further resulted in a reduced synthesis of proteoglycans (PGs) in developing chondrocytes. (2) GC causes cell cycle inhibition by suppressing the promyelocytic leukemia zinc finger (PLZF) gene. (3) Glucocorticoid-induced activation of Bax and its translocation to the mitochondrial membrane leads to subsequent induction of apoptosis. (4) GC causes a decrease in the anti-apoptotic proteins Bcl-2 and Bcl-x in growth plate chondrocytes. (5) GC inhibits KLF2 expression which regulating the Runx2-mediated PI3K/AKT and Erk signaling pathways responsible for the chondrocyte apoptosis.

Glucocorticoids (GCs) are a class of steroid hormones that are produced naturally by the adrenal gland and regulate various physiological processes in the body, including metabolism, immune response, and stress response. The endogenous physiological dose of glucocorticoid (GC) plays a key role in maintaining normal bone metabolism and osteogenic differentiation (1). Synthetic glucocorticoids are widely used in medicine as anti-inflammatory and immunosuppressive agents (2). They can be divided into long-acting, medium-acting and short-acting based on their duration of action and half-life. Short-acting glucocorticoids, such as hydrocortisone and cortisone, are primarily used for replacement therapy of adrenocortical insufficiency (3), while medium-acting glucocorticoids, such as prednisone and methylprednisolone, are mainly used for anti-rheumatic diseases, autoimmune diseases, immune transplantation therapy, etc. (4, 5). Long-acting glucocorticoids, such as dexamethasone and betamethasone, have strong anti-inflammatory potency, long duration of action, and are preferred for anti-allergy(6). Exogenous synthetic GCs are also commonly used in children with progressive muscle dystrophy (7, 8) and respiratory diseases (6). The therapeutic effect of GC has been reported very intensively, however, the side effects particularly in skeletal system caused by long-term or high-dose medication cannot be ignored. Since the skeletal system of adolescents has not yet been developed, GC can induce bone growth suppression, resulting in a significant decrease in height (9). Currently, drugs for the treatment of GC-induced osteoporosis cannot effectively reduce growth suppression(10). Therefore, there is still a lack of specific drugs to tackle GC-induced growth suppression. Recent studies have actively explored the mechanism of GC-induced bone growth retardation, which is a complex process involving multiple pathways (**Figure 1**). GCs affects bone growth mainly through two ways. First, GCs can affect physiological process by regulating hormone (11, 12), growth factors (13), calcium and phosphorus metabolism (14) and angiogenesis (15). Second, GC can affect bone growth in cellular behaviors by inhibiting the chondrocytes in long bone growth plate. GCs can directly inhibit chondrocyte proliferation and differentiation (16, 17), matrix proteoglycan synthesis(18), and cell apoptosis (**Figure 2**) (19). This review summarizes the recent studies on the pathological mechanism of glucocorticoid-induced bone growth retardation, providing valuable insights for the development of targeted therapies to address this issue.

## 2 Advances in pathological mechanism of bone growth disorder in children caused by GC

### 2.1 Effects of GC on endocrine hormones

#### 2.1.1 Growth hormone (GH) and insulin growth factor-1 (IGF-1)

GH is secreted by pituitary cells in the human brain and is mainly regulated by growth hormone-releasing hormones secreted by the hypothalamus. GH exerts its pleiotropic effects through growth hormone receptor (GHR), which can be activated directly by tyrosine kinase activation (20) or indirectly by the induction of IGF-1 (21). Systemic corticosteroids have been shown to inhibit GH secretion (22) and IGF-1 activity (23), and this inhibitory effect is mediated by altering the regulation of somatostatin in the hypothalamus (23). Recent study have demonstrated that dexamethasone (DEX) down-regulates the mRNA expression and binding ability of GHR in growth plate chondrocytes in a dose- and time-dependent manner, and reduces the homologous increase of the IGF-1 receptor (IGFR) and GHR expression (24). In addition, after DEX treatment of pregnant SD rats, several components of the IGF-1 signaling pathway, including IGF-1 receptors, insulin receptor substrates, and serine-threonine protein kinases, were down-regulated in fetal rat growth plate chondrocytes (24). These findings provide insights into the mechanism by which GC interferes with the physiological stimulation of GH and IGF-I on the proliferation of epiphyseal chondrocytes.

#### 2.1.2 Thyroid hormone

Thyroid hormone (triiodothyronine, T_3_) is essential for bone growth after birth by binding to nuclear receptors TRα1 and TRβ1 in chondrocytes and osteoblasts (25). Impaired thyroid function can result in abnormally thin growth plates, impaired chondrocyte hypertrophy, short stature, delayed bone age, skeletal dysplasia, and delayed tooth development in childhood (26, 27). Hypothyroid rats exhibit disordered growth plates and reduced areas of hypertrophy (28), while hyperthyroidism can lead to accelerated ossification of long bones and cartilage (29). Clinical data indicate that in the serum of patients with Cushing’s syndrome, both male and female, thyroid hormone levels and free T3 are lower than those in the normal control group (30). GC can inhibit the expression of parathyroid hormone-related protein (PTHrP) in the growth plate, resulting in growth retardation (19). Studies in adult mice have shown that parathyroid hormone (PTH) can prevent GC-induced osteoporosis, possibly by blocking osteoblast and osteocyte apoptosis (31). Thyroxine (T4) combined with GC treatment can increase the total growth plate length and restore GC-induced growth inhibition (32).

#### 2.1.3 Estrogen

Estrogen is primarily secreted by the ovaries or produced by the conversion of male hormones by aromatase. The regulation of estrogen on long bone is mainly manifested in two aspects, either through a synergistic effect with other hormones such as GH (33), or by directly binding to estrogen receptors (ERs) to control the physiological process of long bone growth plate (34, 35). Studies have shown that after GC intervention in rats, estrogen levels tend to decrease (36). Estrogen can regulate osteoblast activity and apoptosis (37, 38), effectively inhibit osteoclast-mediated bone resorption (39), and maintain the balance between osteogenesis and osteoclastogenesis. Insufficient estrogen secretion can disrupt this balance, resulting in growth plate ossification disorder. Therefore, the occurrence of growth and development disorders caused by GC may be related to its synergistic effect with estrogen.

### 2.2 Effect of GC on fibroblast growth factor 23 (FGF23)

Fibroblast growth factor 23 (FGF23) is a hormone synthesized by bone cells that regulates the ‘bone-kidney’ axis and calcium phosphate metabolism (40–42). FGF23 reduces serum phosphate levels by inhibiting proximal tubular phosphate reabsorption and intestinal phosphate absorption (43), while also reducing plasma calcitriol levels by down-regulating the expression of the renal 1-α hydroxylase and up-regulates 24-hydroxylase (44). These mechanisms explain the hypophosphatemia effect of FGF23. FGF receptors are expressed in most tissues including chondrocytes, and regulate their proliferation, differentiation, and mineralization (45–47). GC can up-regulate the expression of FGF23 in bone and plasma, activating FGFR3 receptors and contributing to GC-induced growth disorders *via* the FGF23/Klotho/FGFR3 pathway (48). However, some studies suggest that dexamethasone and prednisolone can down-regulate FGF23 transcription and FGF23 protein synthesis in the osteoblast-like cells and strongly reduce plasma FGF23 concentration in C57BL/6 mice (49).

### 2.3 Effects of GC on Indian hedgehog factor (IHH)

IHH belongs to the hedgehog protein family and is a morphogenetic protein that plays a crucial role in embryonic formation and development. IHH is a regulator of chondrocyte differentiation rate (50) and is necessary for the osteoblast lineage in developing long bones, working in conjunction with other factors (such as BMPs) to induce osteoblast differentiation (51). However, studies have shown that GCs can inhibits the proliferation of growth plate chondrocytes and promote their apoptosis by blocking the Ihh/PTHrP signaling pathway *in vitro*. These findings suggest that GC-mediated IHH disorders may lead to growth plate dysplasia (52).

### 2.4 Effect of GC on C-type natriuretic peptide (CNP)

CNP plays an important role in cartilage growth and endochondral bone growth. CNP and its receptor guanylyl cyclase B (GC-B) are effective stimulators of endochondral bone growth. Cartilage-specific CNP or GC-B knockout mice have significantly shorter bones (53). Loss-of-function mutations in natriuretic peptide receptor (NPR)-B, which encodes for CNP receptor GC-B, have been discovered in extreme dwarfing (54), highlighting the importance of CNP in human cartilage growth. CNP promotes bone growth by facilitating the entry of calcium ions into the growth plate chondrocytes through the NPR2-PKG-BK channel and TRPM7 channel-CaMKII axis (55). Studies have shown that high doses of DEX in young male rats significantly reduced the concentration of NT-proCNP, a marker of CNP production (13, 56), and reduced the thickness of the growth plate and bone length.

### 2.5 Effects of GC on vascularization

The epiphysis adjacent to the hypertrophic zone is the site of vascular and osteocyte invasion and longitudinal bone growth. Vascular endothelial growth factor (VEGF) is a crucial angiogenic factor expressed in many tissues and cell types (57), including growth plate cartilage (57, 58). Studies have shown that dexamethasone affects VEGF expression in epiphyseal chondrocytes, and prednisolone treatment in piglets severely disrupts vascular invasion of the growth plates (59). Additionally, GC treatment induces senescence and increases the cell apoptosis of vascular endothelial cells in the metaphysis of the long bones (60, 61). Furthermore, GC treatment severely interferes with VEGF expression in hypertrophic chondrocytes, impairing normal invasion of blood vessels from the metaphysis to the growth plate and bone formation at the cartilage-bone junction. These effects can alter the kinetics of endochondral ossification, leading to GC-induced growth retardation (59, 62).

Recent studies have highlighted the crucial role of vascularization in regulating longitudinal bone growth during endochondral ossification. Romeo et al. reported that proteases released from type H endothelial cells, rather than osteoclasts, are critical for absorbing cartilage and promoting to longitudinal bone growth (63). This suggests that vascular-associated osteoclasts can stimulate endothelial cells to help digest cartilage templates, thereby regulating vascular growth during endochondral ossification. However, Wang et al. found that treatment with prednisolone significantly reduced the number of CD31 and Emcn double-positive H-type vessels in 8-week-old C57BL/6 mice (64). Moreover, dexamethasone inhibited the formation of H-type vessels in the bones of female offspring rats before and after birth through the PDGFRβ/FAK pathway. *In vitro* administration of high concentrations of dexamethasone also inhibited the angiogenesis of endothelial progenitor cells. These findings suggest that GCs can inhibit the development of H-type blood vessels in the bone tissue, leading to the inhibition of long bone development (15). Therefore, GCs can have negative effects on vascularization and angiogenesis, which are essential for normal bone growth and development.

### 2.6 Direct effect of GC on growth plate chondrocytes

As one of the main components of cartilage matrix, proteoglycans (PGs) play an important role in cartilage formation, matrix stability and cell proliferation during early embryonic development (65, 66). Uridine diphosphate glucose dehydrogenase (UGDH) is a key enzyme in the synthesis of PGs in various cell types and involved in maintaining the articular cartilage homeostasis and the development of osteoarthritis. Silencing UGDH gene with specific siRNA significantly reduced the PGs content in human chondrocytes (67). Studies have shown that rat maternal exposure to dexamethasone induces GR to directly bind to the UGDH promoter in fetal rat growth plate chondrocytes, recruiting HDAC1 and Sp3, inducing H3K9 deacetylation, and inhibiting UGDH gene expression. These results in reduced PGs synthesis in developing chondrocytes, leading to disrupted fetal long bone development (18). Therefore, GCs have negative effects on PG synthesis, which is essential for normal cartilage and bone development.

Chondrocytes are initially derived from limb mesenchyme during embryonic development and are located in three different areas of the growth plate: quiescent zone, proliferative zone and hypertrophic zone (68). The quiescent zone contains self-renewing, slowly proliferating chondrocytes that produce highly proliferating chondrocytes that form columns along the bone axis and form a proliferative zone (69). Growth plate senescence is caused by the qualitative and quantitative consumption of stem cell-like cells in the quiescent zone, excessive GC can preserve the proliferative capacity of the growth plate by slowing down the proliferation rate of chondrocytes in the quiescent zone and the consumption of these cells (70). GC treatment of precartilaginous cell line ATDC5 inhibited cell cycle and caused the chondrocyte growth arrest (71). Knockdown of Plzf gene by shRNA alleviated GC-induced cell cycle arrest. This explains the phenomenon of long bone catch-up growth after GC treatment cessation (72, 73). The growth of the long bone is the result of continuous downward proliferation and differentiation of growth plate chondrocytes. Different *in vitro* and *in vivo* studies have revealed the strong inhibitory effect of DEX on chondrocyte proliferation (16, 74). Dexamethasone inhibits KLF2 expression in rat tibial growth plate chondrocytes and promotes dexamethasone-induced proliferation inhibition and its apoptosis by targeting the Runx2-mediated PI3K/AKT and Erk signaling pathways (75). DEX acts in a gene-specific manner in cartilage. It promotes the expression of extracellular matrix (ECM) and metabolic transcripts necessary to maintain the phenotype of chondrocytes, and down-regulates cytokines and growth factors that stimulate cartilage to bone transformation (76). Therefore, GC-treated ATDC5 cells showed reduced nodule formation, no alkaline phosphatase (ALP) and alcian blue-positive ECM and matrix mineralization (77). The above results suggest that GC has the effect of inhibiting proliferation, differentiation, and matrix mineralization of growth plate chondrocytes.

Chondrocyte apoptosis is closely related to systemic GC treatment. The application of dexamethasone in rats can lead to apoptosis by activating caspase-3 in the three regions of the growth plate (75). *In vitro* DEX treatment of HCS-2/8 chondrocytes resulted in a significant increase in apoptosis, which was due to increased caspase-3 cleavage and activation of caspase-8 and -9 by cleavage of the pro-apoptotic factor Bid (78). In addition, DEX induced Bax activation and translocation to the mitochondrial membrane, which subsequently induced apoptosis (79, 80), protecting Bax-deficient mice from dexamethasone-induced apoptosis and growth retardation of growth plate chondrocytes (79). At the same time, the anti-apoptotic proteins Bcl-2 and Bcl-x in each layer of growth plate chondrocytes were significantly reduced after GC treatment (19). Therefore, anti-apoptotic proteins and pro-apoptotic proteins of the Bcl-2 family may be the key factors of DEX-induced growth plate chondrocytes apoptosis (81).

### 2.7 Effect of GC on hyperactivation of osteoclasts

GCs have been shown to have significant effects on osteoclasts during bone growth. GCs stimulate osteoclast differentiation and activity, leading to increased bone resorption and decreased bone formation. This can result in reduced bone mineral density and increased risk of fractures, particularly in children and adolescents who are still growing (82, 83). GCs alter the expression of genes involved in osteoclast differentiation and activity, including RANKL, OPG, and NFATc1 (84). GCs activate osteoclasts *via* secondary hyperparathyroidism and enhance the maturation and activation of osteoclasts. GCs can both increase osteoclast lifespan by inhibiting osteoclast apoptosis and induce increased osteoclastic bone resorption (85). Recent studies highlight a crucial role of reactive oxygen species (ROS) in osteoclast formation and function by modulating receptor activator of NF-κB ligand (RANKL)-induced signaling, which eventually leading to osteoclast hyperactivity in GC-induced osteonecrosis of the femoral head (ONFH) (86), suggesting antioxidant therapy as a potential alternative to prevent GC-induced ONFH by suppressing ROS level and thereby inhibiting osteoclasts. Additionally, GC may exert diverse effects on osteoclasts when used for the treatment of inflammatory diseases. For example, temporal use of GCs may relieve rheumatoid arthritis (RA) progress, while long-term use of GCs may lead to excessive bone resorption *via* activating osteoclasts, suggesting a central role of immune system in regulating bone metabolism in RA (87, 88).

## 3 Therapeutic strategies for skeletal growth inhibition caused by GC

### 3.1 General treatment precautions

Since GC use is the most common iatrogenic cause of bone growth inhibition in children, but this side effect can be largely prevented. The first step is to minimize the use of oral GC in terms of dose and duration. If GC treatment is considered necessary, prevention of bone growth inhibition should also be considered. For patients who need to use GC, when the condition is stable, priority should be given to the treatment of local, short-term, low-dose and alternate-day administration, and short-acting GC with less inhibitory effect on bone growth should be selected as far as possible (89). A clinical study found that (90), compared with short-term administration once a day in the morning, taking 5 mg prednisolone once in the evening can inhibit 24-hour growth hormone secretion. Therefore, compared with taking exogenous GC at night, taking in the morning has less effect on growth rate. Follow-up of children with severe conditions requiring high-dose GC, especially in the first 1-2 months of initial treatment, the changes of plasma GH, IGF-1, C-type natriuretic peptide, amino-terminal Pro-CNP, blood glucose, insulin, blood calcium and other biochemical indicators can be monitored. Through the analysis and evaluation of endocrinologists, the growth status of children can be determined and vitamin D and calcium should be supplemented routinely (91).

At present, no drug has been approved by the FDA for clinical treatment of bone growth retardation caused by GC. However, there are medications that can be used to help mitigate the effects of glucocorticoids on bone growth. These medications may be prescribed off-label by a healthcare provider to help address the issue of bone growth retardation (**Table 1**).

**Table 1:** Emerging drugs under investigation in clinical trials for bone growth inhibition.

Type of drug	Drug name	Route of administration	Pharmacological action
GH analogue	Recombinant growth hormone	Subcutaneous injection	Supplement growth hormone Inhibit protein metabolism
IGF-I analogue	Recombinant human insulin-like growth factor-1	Subcutaneous injection	Insulin-like growth factor-I supplementation Stimulate chondrocyte hypertrophy
CNP transcription products	C-type natriuretic peptide	Subcutaneous injection	Stimulate CNP receptors on growth plate chondrocytes
Anti-apoptotic protein	Endogenous anti-apoptotic protein humanin	Subcutaneous injection	Block the activation of apoptotic proteins
Traditional Chinese medicine	Nourishing yin and cleaning heat Chinese medicine	Oral	Regulate the expression of growth plate ER-a and IGF-1R
Traditional Chinese medicine	Oysters	Oral	Promote growth hormone (GH) circulation and insulin-like growth factor-1 (IGF-1) expression
Traditional Chinese medicine	Salvia miltiorrhiza	Oral	Protective effect on vascular endothelial cells Improve blood circulation in the uterus and placenta obvious estrogen-like effect
Traditional Chinese medicine	Resveratrol	Oral	Suppress vascularization Delay chondrocyte senescence
Traditional Chinese medicine	Phlomis umbrosa	Oral	Protective effect on vascular endothelial cells Improve blood circulation in the uterus and placenta obvious estrogen-like effect

### 3.2 Listed clinical drugs

#### 3.2.1 Recombinant growth hormone (rhGH)

In addition to inhibiting bone growth, long-term administration of pharmacological doses of GC can also cause muscle atrophy (92). Short-term administration of rhGH to normal volunteers has shown an inhibitory effect of prednisone on acute catabolism of protein (93, 94). Administration of rhGH significantly antagonizes the side effects of long-term GC administration, such as protein consumption, osteoporosis, and hyperlipidemia (95). rhGH can also improve growth of children receive GC treatment after liver transplantation (96). A 5-year prospective open study found that (97) children receiving GC treatment, after 36 months, rhGH group height standard deviation score change preliminary analysis significantly increased by (0.80 ± 1.03), but two patients experienced treatment-related adverse reactions: one case with poor compliance, the other case with mild hyperglycemia. Studies have shown that rhGH therapy is effective in increasing height in children with long-term GC treatment and is tolerable. However, many potential risks are still existing in rhGH treatment, including undetermined risks such as increased intracranial pressure, malignant tumors, femoral head spondylolisthesis, insulin resistance, and type 2 diabetes, which limit its large-scale use. More preclinical studies are still needed to verify the safety and efficacy of rhGH in GC-induced growth disorders (98).

#### 3.2.2 Recombinant human insulin-like growth factor-1

Insulin-like growth factor-I (IGF-I) is naturally produced by many tissues, including liver and skeletal muscle. It is the main mediator of growth hormone (GH) for normal bone growth and is also important for muscle cell regeneration and survival (99, 100). In preclinical studies, rhIGF-1 is beneficial in animal models of muscle injury, wasting and aging (101, 102). Clinically, rhIGF-1 is approved for the treatment of growth disorders in children with severe primary IGF-I deficiency (103, 104). Studies have shown that DEX impairs longitudinal growth by inhibiting chondrocyte proliferation, while IGF-I can stimulate chondrocyte hypertrophy and reverse the inhibitory effect of DEX on growth (105). Claire L Wood et al. used growth hormone and insulin-like growth factor-1 to rescue the growth retardation of x-linked muscular dystrophy (mdx) mice treated with GC (106).

### 3.3 Unlisted drugs

#### 3.3.1 C-type natriuretic peptide (CNP)

As mentioned above, CNP transcripts and their receptor NPR-B are expressed in chondrocytes of growth plates (56). Daily subcutaneous injection of CNP-53 can activate bone growth and reverse the growth inhibition caused by GC treatment in mice (107). At present, CNP-like drugs have entered phase II clinical trials in humans (108–110). Preliminary data show that recombinant CNP is safe for children and can improve the growth rate of children with chondrodysplasia. Up till now, there are no adverse reactions and allergic reactions related to bone growth. This data shed light on the clinical application of CNP in the treatment of GC-induced growth disorders.

#### 3.3.2 Endogenous anti-apoptotic protein humanin (HN)

HN is a polypeptide composed of 24 amino acids and was originally found to be a neuroprotective factor (111). HN has also been reported to exert anti-inflammatory (112) and anti-apoptotic effects by blocking the activation of pro-apoptotic proteins Bax (113) and Bak (114). HN treatment has shown promising results in preclinical models of diabetes (115), stroke(116), atherosclerosis(117) and Alzheimer’s disease(118). HN analogues (HNG) prevent bone growth retardation, chondrocyte apoptosis and proliferation inhibition by up-regulating the Hedgehog pathway without interfering with the anti-inflammatory effects of DEX(119).

### 3.4 Chinese medicine on GC growth inhibition treatment prospects

According to traditional Chinese medicine theory, the kidney as the body’ s innate foundation, is also the root of the five organs of yin and yang, namely ‘yin and yang secret, spirit is the rule’. Yin and yang balance is fundamental to maintain normal physiological activities. GC is a kind of hormone secreted by adrenal cortex, which can be regarded as the nature of ‘pure yang’. GC under physiological dose has the meaning of ‘less fire generates qi’. In the case of long-term use of exogenous hormones beyond physiological dose, this kind of ‘pure yang’ medicine is easy to ‘overcome yang and consume yin’, which affects the introversion of yin essence and cannot play its nourishing role, resulting in kidney yin deficiency. When the human body are weak for a long time, it is easy to produce blood stasis, which hinders the generation and distribution of fresh blood, and reacts on deficiency, deficiency, and blood stasis, which accumulating in the body, disturbing the body’s qi, blood, yin and yang, and finally causes metabolic disorders. These in turn affect the nutrition and metabolism of the body organs(120, 121). Improving the body’s nutritional metabolism and blood stasis may have a certain preventive effect on the side effects of GC. Clinical use of traditional Chinese medicine can slow down the heat and yin caused by a large number of GC symptoms and signs, kidney strengthening medicine can promote growth and development and make children grow taller (122). Studies have shown that nourishing yin and purging fire Chinese medicine regulates bone development and maturation by regulating the expression of growth plate ER-α and IGF-1R (123). Shen Huansi et al. utilized Shengdi and tortoise shell, the traditional Chinese medicine for nourishing yin and clearing heat, to antagonize the growth retardation induced by dexamethasone in rabbits, possibly by regulating IGF-1 in the growth plate to reduce the inhibitory effect of DEX on the growth plate (124). Traditional Chinese medicine believes that oysters are beneficial to yin and yang, astringency and astringency to preserve kidney essence (125). According to the study, fermented oysters rich in γ-aminobutyric acid (GABA) promoted growth hormone (GH) circulation and insulin-like growth factor-1 (IGF-1) expression in young rats (126, 127), and increased the height and body length of the growth plate. Salvia miltiorrhiza is a commonly used traditional Chinese medicine for promoting blood circulation and removing blood stasis (128). Studies have found that *Salvia miltiorrhiza* has a protective effect on vascular endothelial cells in rats with blood stasis syndrome (129, 130). Clinical studies have found that Danshen can also treat pregnancy with fetal growth restriction (FGR), possibly by improving blood circulation in the uterus and placenta (131). At the same time, Danshen also has obvious estrogen-like effect, making it a potential alternative to estrogen in treating skeletal system disorders caused by estrogen reduction (132). Resveratrol is a natural antioxidant, which plays an important role in orthopedic diseases such as osteoarthritis (133), osteoporosis (134) and nerve injury repair (135). Resveratrol has been found to have the potential to improve longitudinal bone growth, which is associated with delayed growth plate fusion, resulting in increased final length (136). At the same time, resveratrol also has the effect of inhibiting the apoptosis of growth plate chondrocytes and delaying epiphyseal closure (137). Phlomis umbrosa has the functions of detumescence, muscle growth, tendon continuation and bone grafting. It was found that the mixture of Phlomis umbrosa, Astragalus membranaceus and Acanthopanax senticosus could increase the longitudinal bone growth rate of growing rats, improve the quality of bone trabeculae, and enhance the microstructure of bone trabeculae and cortical bone during growth (138). Donghun Lee et al.used the extract of Phlomis gracile to treat female adolescent rats and found that it increased the longitudinal bone growth rate and promoted the proliferation and differentiation of chondrocytes by up-regulating the expression of local IGF-1 and BMP-2 in the growth plate (139).

In summary, Chinese medicine has a comprehensive, multi-targeted and broad-spectrum effect in promoting bone growth. It can promote bone growth by affecting the GH-IGF axis, inhibiting the apoptosis of growth plate chondrocytes and delaying the closure of the growth plate. Although Chinese medicine has significant effects on promoting bone growth and development, the current studies are mostly animal experiments and lack of standardized clinical trials. In addition, compound formulas have variable effects and complex mechanisms, and the current research on single drug is mainly based on its extracts. Future studies should focus on the elaboration of multi-target Chinese medicine treatment from molecular biology and cell biology, combined with multicenter, large sample, double-blind, randomized controlled clinical studies.

## 4 Conclusion

This systematic review provides a comprehensive summary of recent research progress in the mechanism of GC-induced growth suppression in children. Long-term application of GCs have negative effects on the systemic endocrine system and local long bone growth plate, leading to growth suppression. Currently, there are no effective drugs to combat the negative effects of GC, and only rational application of GC and nutritional supplements are available for prevention. The complexity of its mechanism of affecting growth disorders requires further explorations and efforts to dissect its exact molecular mechanism. In conclusion, the most effective prevention and treatment method of GC-induced growth disorders remains rational GC use. Off-label use of medications, such as rhGH, may be prescribed to help address the bone growth retardation, but additional clinical trials are needed to verify their safety and efficacy.

## Author contributions

JYH and SL carried out the literature review and drafted the manuscript. JYH, JPH, and SL revised the manuscript. SL, GL, and LC finalized and approved the manuscript for submission and provided funding support. All authors contributed to the article and approved the submitted version.
